# Pattern Transition on Inertial Focusing of Neutrally Buoyant Particles Suspended in Rectangular Duct Flows

**DOI:** 10.3390/mi12101242

**Published:** 2021-10-14

**Authors:** Hiroshi Yamashita, Takeshi Akinaga, Masako Sugihara-Seki

**Affiliations:** 1 Department of Pure and Applied Physics, Kansai University, 3-3-35 Yamate-cho, Suita, Osaka 564-8680, Japan; sekim@kansai-u.ac.jp; 2 Department of Systems Design Engineering, Akita University, 1-1 Tegatagakuen-Machi, Akita 010-8502, Japan; akinagat@gipc.akita-u.ac.jp

**Keywords:** particle-laden flow, particle-focusing phenomenon, inertial lift force

## Abstract

The continuous separation and filtration of particles immersed in fluid flows are important interests in various applications. Although the inertial focusing of particles suspended in a duct flow is promising in microfluidics, predicting the focusing positions depending on the parameters, such as the shape of the duct cross-section and the Reynolds number (Re) has not been achieved owing to the diversity of the inertial-focusing phenomena. In this study, we aimed to elucidate the variation of the inertial focusing depending on Re in rectangular duct flows. We performed a numerical simulation of the lift force exerted on a spherical particle flowing in a rectangular duct and determined the lift-force map within the duct cross-section over a wide range of Re. We estimated the particle trajectories based on the lift map and Stokes drag, and identified the particle-focusing points appeared in the cross-section. For an aspect ratio of the duct cross-section of 2, we found that the blockage ratio changes transition structure of particle focusing. For blockage ratios smaller than 0.3, particles focus near the centres of the long sides of the cross-section at low Re and near the centres of both the long and short sides at relatively higher Re. This transition is expressed as a subcritical pitchfork bifurcation. For blockage ratio larger than 0.3, another focusing pattern appears between these two focusing regimes, where particles are focused on the centres of the long sides and at intermediate positions near the corners. Thus, there are three regimes; the transition between adjacent regimes at lower Re is found to be expressed as a saddle-node bifurcation and the other transition as a supercritical pitchfork bifurcation.

## 1. Introduction

The transport of objects immersed in fluid flows through a duct and the technique to separate them are important for applications in biology, medicine, and industry. Therefore, many studies have been conducted to understand the interaction between an object and a fluid flow and the resulting motion of the object. In this study, we focused on the “tubular pinch effect” [1], which is one of the various fluid dynamic interactions that have been revealed to date. When a spherical particle is neutrally suspended in a laminar flow through a straight duct, the particle moves in the main flow direction and also crosses streamlines because it experiences “inertial lift force” [2]. The lift pointing away from the duct wall is exerted on a sphere located near the wall, whereas the lift in the direction towards the wall occurs when the particle is near the centreline of the duct. At an equilibrium position where the net lift force acting on a particle vanishes, the lateral migration of a suspended particle stops and then the particle moves only in the main flow direction; that is, it focuses on a certain position in the cross-section. The focusing position corresponds to a stable equilibrium position.

Segré and Silberberg [3] first discovered experimentally this particle-focusing phenomenon in the 1960s. It was shown that rigid spherical particles entering randomly through the inlet of a circular tube are focused on an annulus at the downstream cross-section, which is the so-called “Segré–Silberberg annulus”. This particle motion crossing streamlines has attracted a great deal of attention and theoretical analyses have been carried out intensively on the forces acting on a particle immersed in a fluid [4,5].

In the 2000s, Di Carlo et al. [6] reported that particles flowing in a rectangular duct concentrate at several points in the downstream cross-section. The particle-focusing points appeared near centres of the sides of the cross-section. This report suggests the possibility of realising a technique that can separate objects simply by flowing a suspension through a duct. This technology is expected to be particularly useful in advanced medical applications because it is effective for separating objects that are easily damaged by strong external forces such as centrifugation. However, the elucidation of the particle-focusing phenomena due to the tubular pinch effect in rectangular ducts is still insufficient. The reason for this is the diversity of the particle-focusing phenomena.

Among them, the particle focusing in square duct flows has been relatively well clarified in a wide range of Reynolds numbers (Re=ρUh/μ, ρ: fluid density, *U*: maximum flow velocity, *h*: duct width, μ: fluid viscosity). Miura et al. [7] performed the ‘en-face’ observation of particles neutrally suspended in flows through a square duct and showed that at relatively high Re (≈1000), particles are focused near not only centres of the duct sides but also near corners of the cross-section. Nakagawa et al. [8] reproduced the experimental results of Miura et al. [7] by a corresponding direct numerical simulation. A following experimental study indicated the emergence of another type of particle-focusing point at moderate Re (≈700) [9]. From these studies, we can summarise the focusing points of rigid spherical particles in square duct flows and their variation with Re for a blockage ratio d/h≈0.1, where *d* is a particle diameter, as follows. At low Re (⪅400), particles focus near the centres of the duct faces. As Re increases, additional focusing points emerge between the diagonals of the cross-section and centrelines through the midpoints of the sides. This type of focusing does not occur at relatively high Re (≈1000), and the focusing points near the duct corners appear instead. These variations of the particle-focusing points with Re were numerically studied to elucidate the structure of the transitions between the particle-focusing patterns, and the emergence and disappearance of particle-focusing points were explained as bifurcation phenomena in terms of particle equilibrium positions [10].

For rectangular ducts with an aspect ratio of the cross-section A=b/h>1 (*b*: long side width, *h*: short side width), there are different patterns of particle focusing because of the lack of symmetry. Bhagat et al. [11] showed that suspended particles are aligned along the long sides of the duct cross-section at low Re. Harding et al. [12] predicted two types of focusing patterns using the perturbation expansion in the particle Re, that is, at quite low but finite Re. They reported that for A=2, particle-focusing points appear near the centres of long side walls at the blockage ratio d/h=0.15, and near both the centres of the long and short side walls at d/h=0.05, independent of Re. Zhou and Papautsky [13] demonstrated experimentally that particles focus near the centre of the long side of the cross-section for A=2, d/h≈0.15, and Re⪅90. They also showed the two-stage migration property of particles suspended in rectangular duct flows. Firstly, particles entering randomly the duct inlet align along the long side of the cross-section. Secondly, they approach and focus near the centres of the long side. On the other hand, Ciftlik et al. [14] reported the change of focusing patterns depending on Re: for A=1.6 and d/h=0.2, particles are focused near the centres of the long side at low Re whereas they are focused near the centres of both the long and short sides at elevated Re. Liu et al. [15] indicated the presence of these two types of focusing patterns by a direct numerical simulation. They calculated the inertial lift force acting on a spherical particle flowing in rectangular ducts and estimated the particle trajectories based on the lift obtained. In particular, in the case of A=2, they showed a single focusing near the centres of the long sides at Re=100 and the double focusing near the centres of the long and short sides at Re=200 for a blockage ratio of 0.3. In contrast, for a blockage ratio of 0.1, they reported double focusing at both Re=100 and 200. Including these cases, they investigated the particle-focusing patterns for various parameters; however, the structure of the transition between these two focusing patterns remains to be clarified.

In this study, we aimed to elucidate the structure of the transition between the particle-focusing patterns that appear in the suspension flows through a rectangular duct. For A=2 and various blockage ratios, we performed a numerical simulation of the inertial lift force acting on a neutrally buoyant spherical particle immersed in rectangular duct flows and constructed a cross-sectional map of the lift forces. Based on the lift-force map obtained, we determined the particle equilibrium points appearing in the cross-section and estimated the particle trajectories. The focusing patterns of particles suspended in rectangular duct flows were investigated for a wide range of Re. Herein, we elucidate the transition between the focusing patterns as a bifurcation phenomenon in terms of the particle equilibrium points by capturing the Re changes of the nullclines for the particle trajectories.

## 2. Numerical Method

We considered the motion of a neutrally buoyant spherical particle flowing through a rectangular duct with an aspect ratio of its cross-section A=b/h. The governing equations are the continuity equation and Navier–Stokes equations for the incompressible Newtonian fluid flow, and Newton’s second law of motion for the suspended particle. We assumed that the particle moves only in the streamwise direction and it is fixed in the lateral direction, and it freely rotates in all directions. We calculate the lift force in the *x*- and *y*-directions acting on the particle in the steady state, that is, when the particle experiences no drag and torque.

**Figure 1:**
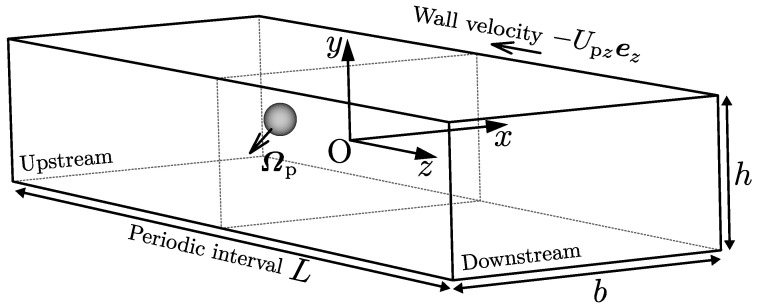
Flow configuration. Galilean transformation in the streamwise direction is adopted.

In this study, the immersed boundary method [16] based on the finite difference method was adopted to numerically solve this problem. We consider the centre of the duct cross-section, where a suspended particle exists, as the origin. The *x*- and *y*-axes are parallel to the long and short sides of the cross-section, respectively, as shown in Figure 1. The *z*-axis is along the centreline of a rectangular duct. The governing equations for the fluid flow are described as below:

(1) ∇·u=0,

(2) $$\rho \left[\frac{\partial u}{\partial t}+\left(u\cdot \nabla \right)u\right]=-\nabla p+\mu \nabla^{2}u-\rho \frac{d}{dt}\left(U_{pz}e_{z}\right)+f,$$

where ρ and μ are the fluid density and viscosity, respectively, and $U_{pz}$ is the translational velocity of the particle. In Equations (1) and (2), the velocity u represents the volume-weighted average velocity that combines the fluid velocity $u_{f}$ and the velocity inside a suspended particle:

(3) $$u=\left(1-\alpha \right)u_{f}+\alpha \left(\Omega_{p}\times r_{p}\right),$$

where $\Omega_{p}$ is the particle angular velocity, and $r_{p}$ is a position vector that starts from the particle centre. The scalar function α depends on $r_{p}$ and represents the volume fraction of the solid phase. It is defined as

(4) $$\alpha =\frac{1}{2}\left(1-\tanh\frac{|r_{p}|-d/2}{\delta_{\alpha }}\right),$$

using particle diameter *d* and parameter $\delta_{\alpha }$ related to the transition thickness between the fluid and solid phases. Following previous studies [8,10], we set $\delta_{\alpha }=0.03d$ which corresponds to 0.6$\delta_{x}$, where $\delta_{x}=0.05d$ and is the width of computational cells. The f in (2) represents the interaction between the fluid and the particle, expressed as the body force exerted on the fluid by the particle:

(5) $$f=\frac{\rho \alpha }{\delta_{t}}\left(\Omega_{p}\times r_{p}-\hat{u}\right)$$

where $\delta_{t}$ is a time increment. $\hat{u}$ represents the temporal velocity obtained when the whole domain is assumed to be in the fluid region:

(6) $$\hat{u}=u^{n}+\delta_{t}{\left[-\left(u\cdot \nabla \right)u-\frac{1}{\rho }\nabla p+\frac{\mu }{\rho }\nabla^{2}u-\frac{d}{dt}\left(U_{pz}e_{z}\right)\right]}^{n}$$

where *n* represents the time-step count. We calculated this temporal velocity by the fractional step method and then used the second-order central difference for the spatial discretisation. The equations of motion for the particle of mass $M_{p}=\rho \pi d^{3}/6$ and moment of inertia $I_{p}=M_{p}d^{2}/10$ are as follows:

(7) $$M_{p}\frac{d}{dt}U_{pz}=-\int_{V}f_{z}dV,$$

(8) $$I_{p}\frac{d}{dt}\Omega_{p}=-\int_{V}r_{p}\times fdV.$$

We numerically solved Equations (7) and (8) by the Crank–Nicolson method. The region *V* corresponds to the computational domain. In the steady state, we determine the lift force $F_{p}$ defined as

(9) $$F_{p}=-\int_{V}\left(f_{x}e_{x}+f_{y}e_{y}\right)dV.$$

We adopted a no-slip boundary condition along the duct side walls. We also adopted the periodic boundary condition between the upstream and downstream cross-sections and then considered the point symmetry in the cross-section:

(10) $$\begin{array}{cc} u(x,y,-\frac{L}{2}) & =u(-x,-y,\frac{L}{2}), \end{array}$$

(11) $$\begin{array}{cc} v(x,y,-\frac{L}{2}) & =-v(-x,-y,\frac{L}{2}), \end{array}$$

(12) $$\begin{array}{cc} w(x,y,-\frac{L}{2}) & =-w(-x,-y,\frac{L}{2}). \end{array}$$

The fluid flow was driven by a constant pressure difference $\delta_{p}$ between both cross-sections:

(13) $$p\left(x,y,-\frac{L}{2}\right)=p\left(-x,-y,\frac{L}{2}\right)+\delta_{p}.$$

Here, we set the periodic interval L=15d. The interval *L* represents the axial distance between adjacent particles. They are located alternatively on the opposite side of the duct centreline, so that they are separated by *L* or more. This configuration is useful compared with that of standard periodic condition because rear particles are less likely to be placed within the wake of front particles when they are located off-centre. We compared the numerical results obtained under the present boundary condition and the corresponding results obtained under the standard periodic condition, for various axial spacing *L*. In the case of A=2, d/h≈0.31, and Re=100, we calculated the lift forces at several lateral positions for L=10d, 15d, 30d under these two boundary conditions, and their values were compared with the lift force for L=45d. For L=45d, the lift forces obtained under the two boundary conditions agreed with each other. The maximum differences were 1.56%, 1.06%, and 0.269% for L=10d, 15d, and 30d, respectively, under the present boundary condition, whereas the corresponding differences were 2.20%, 1.10%, and 0.270% under the standard boundary condition. This comparison confirmed that the results obtained under the present boundary condition are closer to the results for larger axial spacing, compared to those under the standard boundary condition. Considering that the difference was up to about 1% for L=15d, we adopted the present periodic boundary condition with L=15d in this study.

We obtained the lift-force map in the duct cross-section by calculating the lift force acting on a particle which is located at various lateral positions. The locations $\left(x_{i},y_{j}\right)$ are given by

(14) $$\begin{array}{cc} x_{i} & =X_{\max}\xi_{i}=\frac{1}{2}\left(b-\frac{7d}{10}\right)\xi_{i}\;\left(i=0,1,\cdots ,m_{x}\right), \end{array}$$

(15) $$\begin{array}{cc} y_{j} & =Y_{\max}\eta_{j}=\frac{1}{2}\left(h-\frac{7d}{10}\right)\eta_{j}\;\left(j=0,1,\cdots ,m_{y}\right), \end{array}$$

where $\xi_{i}=\cos\left(i\pi /m_{x}\right)$ and $\eta_{j}=\cos\left(j\pi /m_{y}\right)$, called the Gauss–Lobatto points. $X_{\max}$ and $Y_{\max}$ represent the computational ranges of the particle centre position in the *x*- and *y*-direction, respectively. The lift force $F_{p}\left(x,y\right)$ was approximately expressed by an interpolation in terms of the lift force $F_{pij}$ calculated at $\left(x_{i},y_{j}\right)$ as

(16) $$F_{p}\left(x,y\right)=\sum_{i=0}^{m_{x}}\sum_{j=0}^{m_{y}}F_{pij}H_{i}^{m_{x}}\left(x/X_{\max}\right)H_{j}^{m_{y}}\left(y/Y_{\max}\right).$$

Here, the interpolation polynomials $H_{k}^{m}$ are defined as

(17) $$H_{k}^{m}\left(\zeta \right)=\frac{2}{m}\sum_{l=0}^{m}\frac{1}{c_{k}c_{l}}T_{l}\left(\zeta_{k}\right)T_{l}\left(\zeta \right)\;\left(k,l=0,1,\cdots ,m\right),$$

where $\zeta_{k}=\cos\left(k\pi /m\right)$, $c_{n}=1$ for 0<n<m, $c_{n}=2$ for n=0,m, and $T_{n}$ are the Chebyshev polynomials of the first kind. We set $m_{x}=16$ and $m_{y}=14$ following the verification performed in our previous study [10]. From symmetry consideration, we calculated the lift forces at 72 points in the first quadrant of the cross section; that is, $0\leq i\leq m_{x}/2=8$ and $0\leq j\leq m_{y}/2=7$.

To determine the locations where suspended particles focus, the particle trajectories projected over the cross-section were estimated from the lift map obtained. We assumed that a moving particle experiences Stokes drag as the fluid resistance and the lateral migration of a suspended particle is quasi-static. Therefore, the lateral velocity of a suspended particle is described as

(18) $$U_{pi}=F_{pi}/\left(3\pi \mu d\right)\;(i=x,y).$$

Although this assumption is not good enough to estimate the travelling length from the duct inlet needed for particle focusing, the trajectories calculated by Equation (18) are useful to determine the final position where particles focus [10]. Our previous study [10] demonstrated that focusing positions thus estimated agree well with those obtained from the direct calculation of particle trajectories and experimental observations for square duct flows.

**Figure 2:**
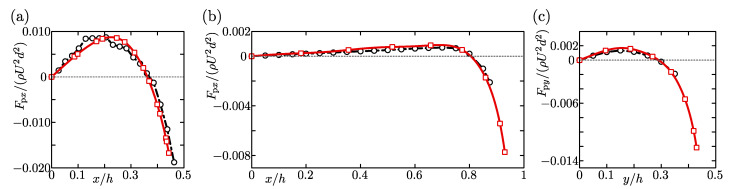
Profiles of the inertial lift force acting on a spherical particle located on the *x*-axis (**a**,**b**) and on the *y*-axis (**c**). The present results are shown by squares. The circles in (**a**) and (**b**,**c**) represent the profiles calculated by Di Carlo et al. [17] and Liu et al. [15], respectively. (**a**) A=1, d/h=0.22, and Re=80, (**b**,**c**) A=2, d/h=0.1, and Re=100.

To assess the accuracy of our numerical simulation, we compared the lift profiles on the axes in the cross-section with those obtained in previous studies [15,17]. Figure 2a shows the profile of the lift force acting on a particle when the aspect ratio A=1 (square duct), the blockage ratio d/h=0.22, and the Reynolds number Re=ρUh/μ=80. Squares and circles in this figure represent our calculations and the results obtained using the finite element method by Di Carlo et al. [17], respectively. Figure 2a indicates that our results are consistent with the lift profile obtained in a previous study. We also calculated the lift forces for the case of A=2, d/h=0.1, and Re=100. The lift profiles on the *x*-axis, which is parallel to the long side of the cross-section, are illustrated in Figure 2b. Figure 2c is the profiles on the *y*-axis. It is found that both the lift profiles on the *x*- and *y*-axes, shown by squares, are in good agreement with circles which represent the profiles calculated by Liu et al. [15]. The numerical method used in the present study was validated for various parameters by comparing our calculations with the previous results.

## 3. Results and Discussion

### 3.1. Two Types of Particle-Focusing Pattern for A=2 and d/h=0.2

**Figure 3:**
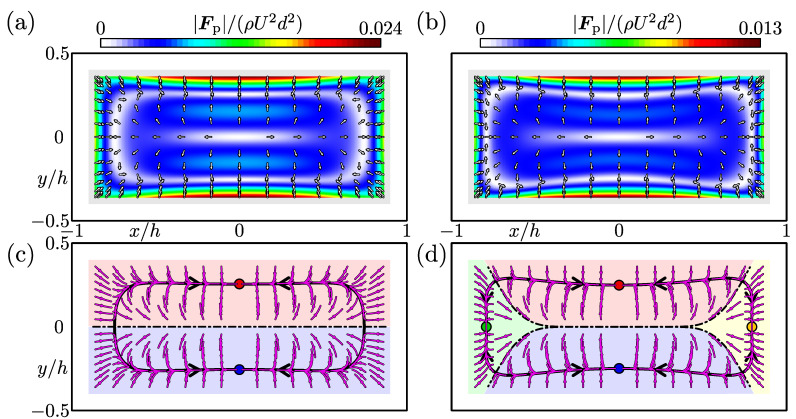
(**a**,**b**) Lift-force maps of the duct cross-section. Each arrow represents the inertial lift force acting on a particle located at its origin. The coloured background is the magnitude of the lift force. The region of existence of the particle centre is depicted by a grey colour in the cross-section. (**c**,**d**) Trajectories of the particle centre projected over the cross-section (curves with arrows) and particle-focusing points (circles). Suspended particles located in each coloured region move towards the focusing point with the corresponding colour. The trajectories drawn by thick curves are called pSS ring [9]. Dash-dotted curves represent the separatrices. A=2, d/h=0.2. (**a**,**c**) Re=50, (**b**,**d**) Re=200.

The inertial-focusing phenomena of a neutrally buoyant spherical particle suspended in rectangular duct flows were numerically investigated for the aspect ratio A=2, various blockage ratios from 0.1 to 0.3 and above, and Reynolds numbers from 50 to 400. In this section, we report our numerical results for A=2 and d/h=0.2, as a representative example. Figure 3a,b show the lift maps for the representative Reynolds numbers Re=50 and 200, respectively. The calculated lift forces are depicted with arrows, and their magnitudes are shown in graded colours in the background. It can be seen from both Figure 3a,b that regardless of the Reynolds numbers, the particles near the side walls experience lift pointing in the opposite direction to the wall, and the lift forces acting on a particle located near the centre of the cross-section are directed towards the side walls.

The particle trajectories estimated from the lift map for Re=50 (Figure 3a) are shown in Figure 3c. Curves with arrows represent the trajectories of the particle centre. This figure indicates the two-stage migration property of particles suspended in a duct flow, which are well-known features of the inertial migration of suspended particles [9,13]. Firstly, particles starting from various positions in the duct cross-section move towards the thick black trajectories depicted in Figure 3c. The closed curve consisting of the thick black particle trajectories correspond to the Segré–Silberberg annulus appeared in suspension flows through a circular pipe, and is, therefore, named “pseudo Segré–Silberberg (pSS) ring” [9]. Secondly, particles migrate along the pSS ring and settle at certain points. In this case of Figure 3c, it is found that particle-focusing points exist near the centres of the long sides of the duct cross-section, as indicated by circles. The dash-dotted line on the *x*-axis in Figure 3c represents the separatrix for the regions in terms of particle-focusing points. The particles initially located inside the region of y>0 (red region) and y<0 (blue region) focus on the points drawn in red and blue, respectively.

Figure 3d shows the particle trajectories and the focusing points obtained for Re=200. The trajectories estimated from the lift map shown in Figure 3b indicate that the pSS ring is slightly deformed compared to that for Re=50, and there are particle-focusing points near the centres of not only the long sides but also the short sides of the cross-section. It was found that the focusing pattern of suspended particles for Re=200 is different from that for Re=50. The existence region of the particle centre, shown in grey in Figure 3b, is divided into four regions by the separatrices. Suspended particles initially located inside each coloured region focus at the position plotted by circles with the corresponding colour. Our calculation suggests that it is relatively difficult to experimentally observe the focusing points near the short sides because the green and yellow regions are smaller than the red and blue regions.

**Figure 4:**
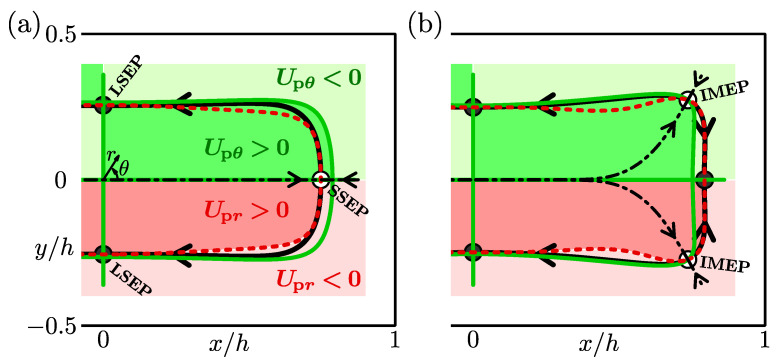
Nullclines of the lateral trajectories of a suspended particle. The red dashed curves represent the zero-level set contour for the radial component of the particle velocity in the cross-section (*r*-nullcline). The green solid curves and lines aligned with the axes are the contours of $U_{p\theta }=0$ (θ-nullcline). Filled and open circles correspond to stable and saddle equilibrium points, respectively. Equilibrium points in (**a**) are the long side equilibrium point (LSEP) and the short side equilibrium point (SSEP), and those in (**b**) are the LSEP, the SSEP, and the intermediate equilibrium point (IMEP). Thick curves with arrows are the particle trajectories and correspond to the pSS ring. Dash-dotted curves represent the separatrices. The regions classified by the sign of $U_{pr}$ are drawn in y<0, whereas the regions in terms of $U_{p\theta }$ are in y>0. $U_{pr}>0$: red, $U_{pr}<0$: light red, $U_{p\theta }>0$: green, and $U_{p\theta }<0$: light green. A=2, d/h=0.2. (**a**) Re=50, (**b**) Re=200.

We have shown two types of particle-focusing patterns at Re=50 and 200 for the aspect ratio A=2 and blockage ratio d/h=0.2. To catch the structure of the transition between these two patterns, the nullclines of the particle trajectories for Re=50 and 200 are illustrated in Figure 4a,b, respectively. We determined the nullclines of the radial and circumferential components of the particle velocity estimated based on the lift-force map and Stokes drag by interpolating the map.

In Figure 4, the red dashed curve shows the zero-level set contour of the radial component of the particle velocity $U_{pr}$, which is named “*r*-nullcline” in the present study. In both Figure 4a,b, $U_{pr}$ is positive inside the *r*-nullcline, whereas it is negative between the nullcline and the side walls. This primarily determines the feature of the first stage for the migration property of suspended particles—particles move towards the pSS ring.

The green solid curve (closed curve in the entire cross-section) and the *x*- and *y*-axes represent the zero-level set contour of the circumferential component of the particle velocity $U_{p\theta }$. We call this contour “θ-nullcline”. The cross-section is divided into eight regions by the θ-nullcline, and the sign of $U_{p\theta }$ changes between adjacent regions, as in the case of the *r*-nullcline. In the first quadrant of the cross-section, the circumferential component of the particle velocity $U_{p\theta }$ is positive (anticlockwise) inside the θ-nullcline (green regions in Figure 4), whereas $U_{p\theta }$ is negative (clockwise) outside it (light green region). This is related to the second stage of the migration property—particles move along the pSS ring and settle at certain points.

The intersections between the nullclines of radial and circumferential motions of the particle, at which $U_{pr}=U_{p\theta }=0$ is satisfied, correspond to the particle equilibrium points because we assume that the particle velocity is proportional to the lift acting on a particle (see Equation (18)). In Figure 4a, for the case of Re=50, the *r*-nullcline and the *x*-axis (the θ-nullcline) intersect. This equilibrium point plotted by an open circle is called “Short Side Equilibrium Point (SSEP)” in the present study. The *r*-nullcline also crosses the *y*-axis (the θ-nullcline). We call this intersection depicted by a filled circle “Long Side Equilibrium Point (LSEP)”. The pSS ring (thick solid curve) consists of heteroclinic orbits joining the SSEP to the LSEP. Arrows depicted on the separatrix (dash-dotted line) and pSS ring indicate that the SSEP is a saddle equilibrium position, whereas the LSEP is stable. Therefore, the particles focus near the centres of the long sides and do not focus near the short sides of the duct cross-section when Re=50 as shown in Figure 3c. Note that the centre of the cross-section is also the equilibrium point. We have found that this point is always unstable; that is, the particles do not focus at the duct centre in the Re range investigated in our study.

The nullclines for Re=200 are shown in Figure 4b. Figure 4b indicates that the particle equilibrium points appear not only on the *x*- and *y*-axes but also near the duct corners. The equilibrium points near the corner (open circles) is named “Intermediate Equilibrium Point (IMEP)”. The emergence of the IMEP is caused by the slight deformation of the nullclines, depending on Re. The *r*-nullcline is located inside the curved θ-nullcline along the side walls when Re=50; that is, they do not intersect (see Figure 4a). As Re increases, both the nullclines are slightly deformed, and a part of the *r*-nullcline near the short side lies outside the curved θ-nullcline. Therefore, the nullclines intersect near the duct corners and the IMEP emerges. The pSS ring for Re=200 consists of heteroclinic orbits connecting the IMEP to the LSEP and the IMEP to the SSEP. The separatrices and pSS ring indicate that the IMEP is a saddle equilibrium point and the LSEP is a stable node. It was also found that the SSEP is changed to be a stable node. At Re=50, the heteroclinic orbit in the first quadrant of the cross-section is located in the $U_{p\theta }>0$ region (green region in Figure 4a) so that the SSEP is a saddle equilibrium point. In contrast, the SSEP is stable when Re=200 because the heteroclinic orbit joining the IMEP to the SSEP lies in the $U_{p\theta }<0$ region (light green region in Figure 4b).

**Figure 5:**
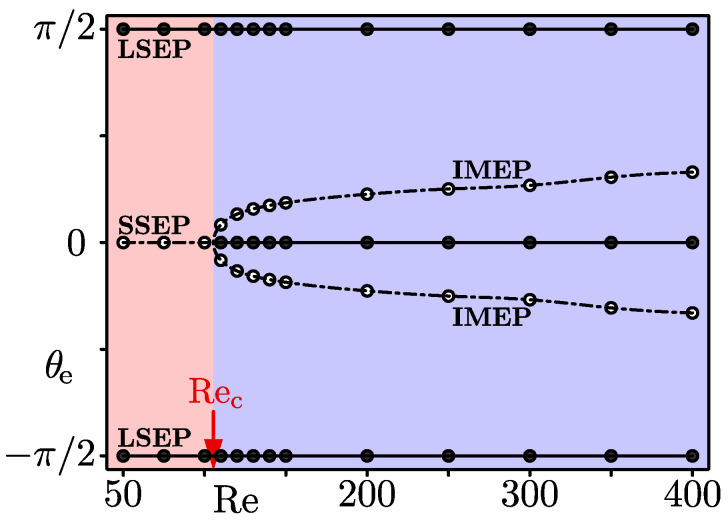
The diagram for the azimuthal angle $\theta_{e}$ of particle equilibrium points. $\theta_{e}=0$: short side equilibrium point (SSEP). $-\pi /2<\theta_{e}<0$, $0<\theta_{e}<\pi /2$: intermediate equilibrium point (IMEP). $\theta_{e}=-\pi /2,\pi /2$: long side equilibrium point (LSEP). The equilibrium points determined from the lift maps are drawn by circles. The filled circles and solid lines represent stable equilibrium points, and the open circles and dash-dotted lines correspond to saddle nodes. The particles focus on the LSEP for Re<Rec (red region), and they focus on the LSEP and SSEP for Rec<Re (blue region). A=2, d/h=0.2.

Figure 5 shows the changes, depending on the Re, of the particle equilibrium points appeared in the first and fourth quadrants of the duct cross-section, that is, in the x≥0 region. We plotted the azimuthal angle of the equilibrium point $\theta_{e}$ as the vertical axis in this diagram. The equilibrium point $\theta_{e}=0$ corresponds to the SSEP, and $\theta_{e}=-\pi /2,\pi /2$ are the LSEPs, and the others are the IMEP. Open circles and dash-dotted lines represent saddle equilibrium points. Filled circles and solid lines represent stable equilibrium points where suspended particles are focused. The diagram indicates that the particles focus at the centres of the long sides (the LSEP) in the Re region with red, whereas they focus at the centres of both the long and short sides (the LSEP and the SSEP) when the Re value is in the blue region. The critical Reynolds number Rec, which corresponds to the border between the red and blue regions, represents the Re value when the transition of the particle-focusing patterns occurs. Our computation showed that the Rec is greater than 100 and less than 110. This value is comparable to the previous estimate Rec≈113 for A=2 and d/h=0.2 by Liu et al. [15]

Our calculation suggests that the pattern transition on the inertial particle focusing for rectangular duct flows for A=2 and d/h=0.2 is explained as a subcritical pitchfork bifurcation. When Re<Rec, the *r*-nullcline lies inside the curved θ-nullclines along the walls, as shown in Figure 4a, so that there are the stable LSEP and the saddle SSEP in the cross-section. At Re=Rec, the *r*-nullcline and the curved θ-nullcline touch each other on the *x*-axis near the short sides. When Re exceeds Rec, a part of the *r*-nullcline near the short sides is outside the curved θ-nullcline, and the SSEP changes from a saddle point to a stable node together with the emergence of the saddle IMEP.

In addition to the case of d/h=0.2, we performed numerical computations for other blockage ratios at A=2. As far as we examined in the rage of 0.1<d/h<0.3, it was inferred that the transition of the particle focusing pattern with Re has the same structure with that for d/h=0.2, i.e., pitchfork bifurcation from the focusing on the LSEP at low Re to that on the LSEP and SSEP at elevated Re.

### 3.2. Three Types of Particle-Focusing Pattern for A=2 and d/h≈0.31

**Figure 6:**
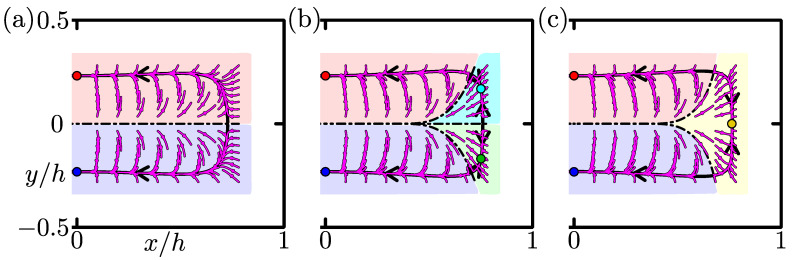
Trajectories of the particle centre projected over the cross-section (curves with arrows) and particle-focusing points (circles). Suspended particles located in each coloured region move towards the focusing point with the corresponding colour. The trajectories drawn by thick curves represents the pSS ring. Dash-dotted curves represent the separatrices. A=2, d/h=0.3125. (**a**) Re=100, (**b**) Re=255, (**c**) Re=300.

**Figure 7:**
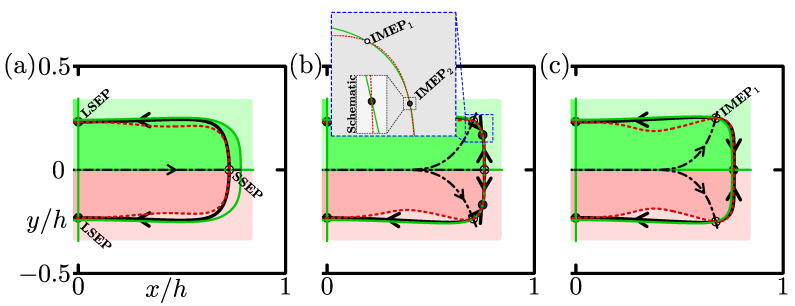
Nullclines of the lateral trajectories of a suspended particle. The red dashed curves represent the *r*-nullcline. The green solid curves and lines aligned with the axes are the θ-nullcline. Filled and open circles correspond to stable and saddle equilibrium points, respectively. Equilibrium points in (**a**) are the long side equilibrium point (LSEP) and the short side equilibrium point (SSEP), those in (**b**) are the LSEP, the SSEP, and two types of the intermediate equilibrium point (IMEP${}_{1}$ and IMEP${}_{2}$), and those in (**c**) are the LSEP, the SSEP, and the IMEP${}_{1}$. A=2, d/h=0.3125. (**a**) Re=100, (**b**) Re=255, (**c**) Re=300.

**Figure 8:**
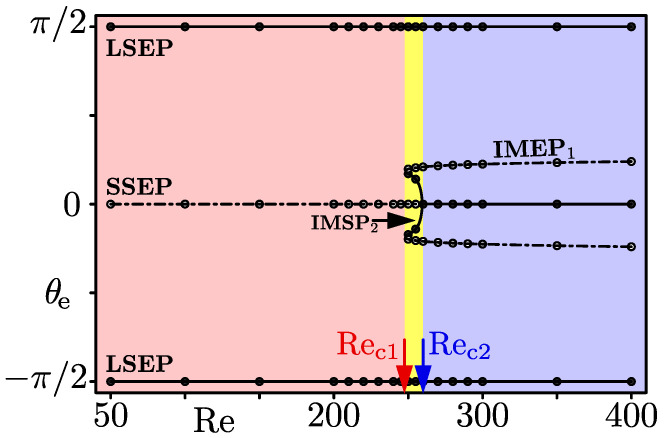
The diagram for the azimuthal angle $\theta_{e}$ of particle equilibrium points. The filled circles and solid lines represent stable equilibrium points, and the open circles and dash-dotted lines correspond to saddle nodes. The particles focus on the LSEP for Re<Rec1 (red region), on the LSEP and the IMEP (IMEP${}_{2}$) for Rec1<Re<Rec2 (yellow region), and on the LSEP and SSEP for Rec2<Re (blue region). A=2, d/h=0.3125.

For d/h⪆0.3, we have found another focusing pattern between the two focusing regimes reported in the previous section. As a representative example, Figure 6, Figure 7 and Figure 8 show our numerical results for A=2 and d/h≈0.31, corresponding to Figure 3, Figure 4 and Figure 5, respectively. In this case, the focusing patterns at a low Re (=100, Figure 6a) and at an elevated Re (=300, Figure 6c) are similar to those for d/h=0.2 at Re=50 and 200, respectively. That is, particles focus on the LSEP at Re=100, and they focus on both the LSEP and SSEP at Re=300. At Re=255 between these Re values, there is a new type of particle focusing pattern as shown in Figure 6b, where particle focusing points are on the LSEP and IMEP near the duct corners. The particle focusing on the IMEP emerges due to crossings of the *r*-nullcline and θ-nullcline near the corner as shown in the inset of Figure 7b. As a result of these crossings, two IMEPs (IMEP${}_{1}$ and IMEP${}_{2}$ in the inset) appear near the long side and short side of the cross-section. The IMEP${}_{1}$ is unstable (saddle point) but the IMEP${}_{2}$ is stable, judging from the particle trajectories shown in Figure 6b. To the authors’ knowledge, this is the first time report of non-LSEP and non-SSEP particle focusing in rectangular duct flows, except for square duct flows. In square duct flows, particle focusing on the IMEP has been observed experimentally and the experimental results were reproduced by numerical simulations [9,10]. Furthermore, the particle focusing on the IMEP was accounted for by crossing of the *r*-nullcline and θ-nullcline, similar to the present study [10]. Our preliminary experiments for rectangular duct flows also indicated the particle focusing on the IMEP for A=1.5 and d/h=0.11, although parameter values are different from the present case [18]. In Figure 7b, relative positions of the *r*-nullcline and θ-nullcline near the SSEP and LSEP are the same with those at low Re (Figure 7a), leading to that their stabilities are the same with those at low Re. Thus, the particle focusing positions are on the LSEP and IMEP at Re=255.

Figure 8 shows a phase diagram of the particle focusing for A=2 and d/h≈0.31. There are two critical Re, Rec1 and Rec2, between adjacent regimes. At Re=Rec1, a saddle-node bifurcation occurs, where the *r*-nullcline and θ-nullcline touch each other. At Re=Rec2, a supercritical pitchfork bifurcation occurs, where the stable IMEP (IMEP${}_{2}$) coincides with the SSEP. To summarise the case of A=2 and d/h≈0.31, the LSEP is stable but the SSEP is a saddle for Re<Rec1; stabilities of the LSEP and SSEP are unchanged but stable and unstable (saddle) IMEPs emerge for Rec1<Re<Rec2; the stable IMEP disappears and the SSEP becomes stable for Rec2<Re.

We showed that for A=2, the transition structure changes depending on whether the blockage ratio is larger or smaller than about 0.3. Harding et al. [12] predicted by a perturbation expansion method that particles would focus on the LSEP and SSEP at low Re for d/h=0.05, suggesting the extinction of the regime where particles focus only on the LSEP. Consequently, there may be another transition structure for small blockage ratios for A=2. Although more detailed study is necessary to elucidate these properties in a wider range of the blockage ratio, we have found that the present method of analysis in terms of *r*-nullcline and θ-nullcline is critical to determine the particle focusing position and to trace the variation of the focusing pattern with parameter values. In addition, the bifurcation diagram in terms of the azimuthal angle of particle equilibrium points is appropriate for intuitive visualisation of the change of the transition structure. The variations in a wide range of d/h are currently under study and the results will be reported in the near future.

## 4. Conclusions

We conducted a numerical simulation of the lift force acting on a neutrally buoyant spherical particle immersed in rectangular duct flows in order to elucidate the structure of the pattern transition on the inertial-focusing phenomena. We constructed the lift-force maps for the aspect ratio of the duct cross-section A=2 and blockage ratios from 0.1 to 0.3 and above in the Re range of 50 to 400. The particle trajectories on the cross-section and the particle-focusing points were estimated based on the lift map obtained and the Stokes drag. From the nullclines of the particle trajectories, we determined the particle equilibrium points and their stability. By tracing the deformation of the nullclines with Re, we found two types of transition structure for the focusing pattern of suspended particles. For blockage ratios smaller than 0.3, we obtained a subcritical pitchfork bifurcation from particle focusing on the LSEP to that on the LSEP and SSEP, while for blockage ratios larger than 0.3, we obtained a saddle-node bifurcation from particle focusing on the LSEP to that on the LSEP and IMEP, followed by a supercritical pitchfork bifurcation to that on the LSEP and SSEP.

## Data Availability

The data presented in this study are available on request from the corresponding author.

## Abbreviations

The following abbreviations are used in this manuscript:

pSS ring	pseudo Segré–Silberberg ring
SSEP	Short side equilibrium point
IMEP	Intermediate equilibrium point
LSEP	Long side equilibrium point
